# Role of Protein Self-Association on DNA Condensation and Nucleoid Stability in a Bacterial Cell Model

**DOI:** 10.3390/polym11071102

**Published:** 2019-06-29

**Authors:** Rita S. Dias

**Affiliations:** Department of Physics, NTNU Norwegian University of Science and Technology, NO-7491 Trondheim, Norway; rita.dias@ntnu.no; Tel.: +47-73593422

**Keywords:** Monte Carlo simulations, H-NS protein, crowding, self-assembly

## Abstract

Bacterial cells do not have a nuclear membrane that encompasses and isolates the genetic material. In addition, they do not possess histone proteins, which are responsible for the first levels of genome condensation in eukaryotes. Instead, there is a number of more or less specific nucleoid-associated proteins that induce DNA bridging, wrapping and bending. Many of these proteins self-assemble into oligomers. The crowded environment of cells is also believed to contribute to DNA condensation due to excluded volume effects. Ribosomes are protein-RNA complexes found in large concentrations in the cytosol of cells. They are overall negatively charged and some DNA-binding proteins have been reported to also bind to ribosomes. Here the effect of protein self-association on DNA condensation and stability of DNA-protein complexes is explored using Monte Carlo simulations and a simple coarse-grained model. The DNA-binding proteins are described as positively charged dimers with the same linear charge density as the DNA, described using a bead and spring model. The crowding molecules are simply described as hard-spheres with varying charge density. It was found that applying a weak attractive potential between protein dimers leads to their association in the vicinity of the DNA (but not in its absence), which greatly enhances the condensation of the model DNA. The presence of neutral crowding agents does not affect the DNA conformation in the presence or absence of protein dimers. For weakly self-associating proteins, the presence of negatively charged crowding particles induces the dissociation of the DNA-protein complex due to the partition of the proteins between the DNA and the crowders. Protein dimers with stronger association potentials, on the other hand, stabilize the nucleoid, even in the presence of highly charged crowders. The interactions between protein dimers and crowding agents are not completely prevented and a few crowding molecules typically bind to the nucleoid.

## 1. Introduction

Prokaryotic cells, such as bacteria, differ from eukaryotic cells in a number of aspects, such as the lack of nuclear membrane. Bacteria genome is thus not physically restricted to a compartment in the cell. In addition, genome packaging does not show the same hyerarchical organization. Bacteria do not possess histones, the class of proteins responsible for the first stages of DNA condensation in eukaryotes [1]. Yet, the genome of *E. coli* K-12, for example, is 4.6 million base pairs long (containing about 4290 protein-coding genes), having a contour length of roughly 1.6 mm, that fits in a cell that is less than 3.0 μm long [2]. It has been shown, using microscopy techniques, that the bacterial genome occupies a well defined region of the cell called the nucleoid [3].

Several mechanisms have been proposed to explain DNA condensation in bacterial cells: nucleoid-associating (DNA-binding) proteins [4], DNA supercoiling, and molecular crowding induced by the presence of a large concentration of macromolecules (e.g., proteins and ribosomes) in the cytosol of bacterial cells [5]. Such large concentrations of macromolecules can lead to attractive depletion forces between or within larger macromolecules [6] and have been suggested to drive the assembly of a wide range of cellular structures [7], including DNA condensation in bacterial cells [8,9]. The synergism of, for example, such forces and DNA-binding proteins have also been explored in the context of DNA condensation in bacterial cells [10,11,12,13,14,15].

The crowded environment is believed to affect the chemical, physical and biological processes within the cells significantly [16]. For example, macromolecular crowding has been shown to affect the diffusion and transport of solutes in solution and within the cell [17], the binding constants of cellular components and reaction rates of e.g., association, site binding, and unfolding [18], and the conformation of different macromolecules [19,20,21,22]. As reviewed in the cited work, the large majority of the studies conducted under crowding conditions use or aim at using so-called inert crowders, chosen so that the interactions between these and the molecules of interest are reduced to exclusion volume effects deriving from the fundamentally impenetrable nature of the molecules. Neutral polymers such as polyethylene glycol (PEG), dextran or Ficoll are often used as crowders, although some debate follows the applicability of PEG as an inert crowding agent [23,24]. However, the macromolecules that can be found in the cytosol of cells do possess charged groups; the presence of charge increases the solubility of the macromolecules and allows for more interaction modes between the components of the cell. Charge-based interactions can be repulsive or attractive, and the charge distribution of groups can also lead to preferential orientations [25,26]. The realization that many of the proteins found in the cell are highly conserved in terms of molecular weight and isoelectric points has led to the description of a fifth level of protein organization, termed the ‘quinary structure’, where the interactions between proteins and other cell components are described as weak and transient [27]. This implies that types of interactions other than steric hard-core repulsions, are likely present between the macromolecule of interest and the crowders in the cell. As recently reviewed [28], non-specific interactions between proteins and crowders have been observed experimentally and using molecular modeling. The presence of non-inert obstacles, mimicking a crowded environment, was found to greatly affect the diffusion of trace particles [29]. Furthermore, the reported protein stabilizing effect that (synthetic) crowders have on proteins has not been found in vivo; some proteins are indeed stabilized in cells but others show no significant effect or are even more prone to conformational changes, when present in the cellular environment [28].

The large majority of the proteins encoded in the genome of e.g., *E. coli* are anionic [30]. The proteins that can be found in larger concentration in the cytosol of the cells are those involved in protein synthesis, most notably ribosomal proteins [31]. These are most often basic with isolectric points in the range 10 to 12, as occurs with other nucleic acid-binding proteins. Ribosomal proteins and RNA associate into ribosomes, which are complexes that overall possess a large negative charge [32]. In fact, the abundance of ribosomes and RNA in the cytosol has been proposed to induce genome condensation and nucleoid formation by a segregative phase separation and demixing between the genome (DNA) and the ribosomes [32]. The presence of negatively charged crowders has been shown to have a large impact on DNA condensation in vitro [33,34,35].

On the other hand, and keeping in mind that the nucleoid is not physically separated from the cytosol by a membrane and that some of the nucleoid-associated proteins bind to DNA with low specificity, it is surprising that the nucleoid is stable and that there is little reported, to our knowledge, on the competition for protein binding between the genomic DNA and the ribosomes and other negatively charged macromolecules in the cytosol. Some classes of proteins primarily identified as DNA-binding proteins (e.g., HU) have some binding affinity to single-stranded DNA and RNA, including that in ribosomes [36,37,38,39]. Regarding non-specific DNA binding agents, it has been shown that heparin, for example, competes with DNA for binding of positively charged species, such as dendrimers [40] and that poly(acrylic acid) prevents DNA condensation by cationic surfactants (Ramisetty, Sovova and Dias, unpublished results).

Different classes of DNA-binding proteins have been identified in bacteria. These are commonly designated architectural proteins due to the structuring effect they have on DNA: they can induce bridging, bending and wrapping [41]. In addition, many of these proteins can oligomerize and be present in cells in polymeric forms [42,43,44,45]. H-NS is an example of such a high-order self-assembling protein [46]. H-NS is a dimer protein and a major component of the nucleoid, and has been shown to affect several processes in the cell, such as gene expression and recombination [47,48]. H-NS binds to DNA with low to medium specificity and has been reported to bind preferentially to AT tracks due to the higher flexibility of the DNA with this composition [49]. Two different binding modes have been reported for H-NS, a bridging mode [50], where the protein forms a bridge between two DNA strands, and a stiffening mode [51], where the H-NS self-assembles along the DNA, leading to its thickening and stiffening. The presence or absence of Mg${}^{2+}$ is believed to dictate the bridging and potential condensation of DNA by the H-NS bridging mode or the extension and stiffening of the DNA, respectively [52]. In addition, protein-protein interactions and protein oligomerization (self-assembly) have been shown to be important for the regulation of gene expression [53,54,55].

In this work, a very simple coarse-grained model is used to describe a bacterial cell composed of a DNA molecule, DNA-binding proteins and molecular crowders. The charge density of the crowding molecules is varied to assess the competitive binding of the DNA-binding proteins to negatively charged crowders. Furthermore, an attractive potential between proteins is applied to explore the effect of protein self-assembly on DNA condensation and the stability of the DNA-protein complex in the presence of crowders.

## 2. Model and Systems

### 2.1. Model

A simple model was adopted to describe a bacterial cell. A 120 monomer-long polyion (model DNA) is described as a sequence of negatively charged hard spheres (monomers) connected by harmonic bonds with the chain flexibility regulated by angular force terms. The nucleoid-associated proteins H-NS form dimers in solution and possess two DNA-binding sites. Electrostatic interactions are very important in DNA–H-NS association; the DNA-binding sites in H-NS have a positively charged surface, given by highly conserved positively charged amino acid residues. In addition, the introduction of negatively charged aminoacids in the DNA binding region has been shown to disrupt the direct interaction of H-NS with DNA [46]. Taking into account that the charge density of the DNA-binding sites in the H-NS and that of DNA is similar [46], we have chosen to ignore the charge distribution and topological properties of both DNA and H-NS and have modelled H-NS simply as two positively charged monomers connected by a harmonic bond (dimer) with the same force constant and equilibrium distance as that of DNA (see below).

The cytoplasm is described as a crowded solution composed of a varying number of spherical particles with radius $R_{\mathrm{crow}}=$ 10 Å, which may or not be charged. All (monovalent) counterions of the DNA, proteins and crowding agents (when charged) are taken explicitly and described as charged hard-spheres with radius 2 Å. The monomers of DNA and H-NS are also 2 Å in radius. All components are enclosed in a spherical cell with radius $R_{\mathrm{cell}}=$ 100 Å, thus the effect of confinement on the conformation of DNA, also occurring in bacterial cells, is included. The solvent enters the model only through its relative permittivity, $\varepsilon_{r}=78.4$, corresponding to that of water at the simulation temperature, T = 298.15 K.

All interactions were taken as pairwise additive. The total potential energy *U* of the system can be expressed as a sum of four contributions according to

(1) $$U=U_{nonbond}+U_{bond}+U_{ang}+U_{ext}.$$

The nonbonded potential energy, $U_{nonbond}$, is given by

(2) $$U_{nonbond}=\sum_{i<j}u_{i,j}\left(r_{i,j}\right)+\sum_{k<l}u_{\mathrm{LJ}}\left(r_{k,l}\right)\;,$$

where the first summation extends over all particles (polyion monomers, simple ions, protein dimers, and crowding agents) with $u_{i,j}$ representing the electrostatic potential plus a hard-sphere repulsion according to

(3) $$u_{i,j}\left(r_{i,j}\right)=\left\{\begin{array}{cc} \infty , & r_{i,j}<R_{i}+R_{j}\\ \frac{Z_{i}Z_{j}e^{2}}{4\pi \epsilon_{o}\epsilon_{r}}\frac{1}{r_{i,j}}, & r_{i,j}\geq R_{i}+R_{j} \end{array}\right.\;,$$

where $Z_{i}$ is the valence of particle *i*, $R_{i}$ the respective radius, $r_{i,j}$ the distance between particles *i* and *j*, *e* the elementary charge, $\epsilon_{o}$ the permittivity of vacuum, and $\epsilon_{r}$ the relative permittivity of the solvent. The second term extends over all protein dimers with $u_{\mathrm{LJ}}$ representing the self-association of the protein dimers calculated according to the Lennard–Jones potential:

(4) $$u_{\mathrm{LJ}}\left(r_{k,l}\right)=4\epsilon_{kl}\left[{\left(\frac{\sigma_{kl}}{r}\right)}^{12}-{\left(\frac{\sigma_{kl}}{r}\right)}^{6}\right]\;,$$

where $\epsilon_{kl}$ is the parameter determining the strength of the attractive potential and $\sigma_{hk}$ the equilibrium distance.

Monomers belonging to the polyion and dimers are connected by harmonic bonds and the bond potential energy of the polyion, $U_{bond}$, is

(5) $$U_{bond}=\sum_{c=1}^{N_{c}}\sum_{i=1}^{N_{mon,c}-1}\frac{k_{bond}}{2}{\left(r_{c;i,i+1}-r_{0}\right)}^{2}\;,$$

where $N_{c}$ is the number of chains (including the DNA and the protein dimers), $N_{mon,c}$ the number of monomers in the chain, $k_{bond}$ = 0.4 Nm${}^{-1}$ is the force constant of the harmonic bond, and $r_{c;i,i+1}$ the distance between two connected monomers, with equilibrium separation, $r_{0}=$ 5 Å.

The angular potential energy, $U_{ang}$, of the DNA chain is given by

(6) $$U_{ang}=\sum_{i=2}^{n-1}\frac{k_{ang}}{2}{\left(\alpha_{i}-\alpha_{0}\right)}^{2}\;$$

where $\alpha_{i}$, are the angles formed by the vectors $r_{i+1}-r_{i}$ and $r_{i-1}-r_{i}$. The equilibrium angle $\alpha_{0}$ = 180${}^{\circ }$ and the force constant $k_{ang}=$ 3.4 × 10${}^{-24}$ J deg${}^{-2}$.

Finally, the confining external potential energy, $U_{ext}$, is given by

(7) $$U_{ext}=\left\{\begin{array}{cc} \infty , & |r_{i}|>R_{cell}\\ 0, & |r_{i}|<R_{cell} \end{array}\right.\;.$$

In this work we have chosen to work with systems where the mixing ratio, defined as $N_{H-\mathrm{NS}}N_{\mathrm{mon},H-\mathrm{NS}}/N_{\mathrm{mon},\mathrm{DNA}}$, is unity and have varied (i) the charge of the crowding particles, $Z_{\mathrm{crow}}=$ 0–−15*e*, corresponding to a surface charge density $\sigma_{\mathrm{crow}}=$ 0–−1.20*e*/nm${}^{2}$; and (ii) the interaction strength of protein self-association $\varepsilon_{\mathrm{pp}}=$ 0–2 kT, where k is the Boltzmann constant.

General data of the model are compiled in Table 1.

### 2.2. Simulation Details

All Monte Carlo (MC) simulations were performed in the canonical ensemble employing the standard Metropolis algorithm [56].

Two different types of MC trial moves were employed for the DNA chains and H-NS dimers, single monomer move and translation of the entire chain. For the model DNA, a slithering move was also used, where a randomly selected end monomer is moved to the opposite end of the chain with biased radial and angular positioning. A schematic representation of the used MC trial moves is shown in Figure S1. The single particle move was attempted 100 times more often than the other types of moves. The crowding agents and counterions were subjected to translational moves.

Each simulation included an equilibration of at least $2\times 10^{6}$ trial moves per particle followed by a production run of at least $6\times 10^{6}$ trial moves per particle. Statistical uncertainties are evaluated by dividing the total simulation into subbatches. All the simulations are performed using the simulation package MOLSIM [57].

## 3. Results and Discussion

In this work, the effect of protein self-assembly in DNA condensation and the stability of the DNA-protein complex was evaluated towards the competitive binding of the proteins to negatively charged crowding agents. These were assessed by calculating the radius of gyration, $R_{G}$, of the DNA according to

(8) $$R_{G}=\frac{1}{N_{\mathrm{mon}}}\sum_{i=1}^{N_{\mathrm{mon}}}\left|r_{i}-r_{\mathrm{CM}}\right|\;,$$

where $r_{i}$ denotes the position of monomer *i* and $r_{\mathrm{CM}}$ the position of the center of mass of the chain.

The interactions between the different components were further evaluated resorting to radial distributions functions, g(r).

### 3.1. Effect of Crowding on DNA Condensation

**Figure 1:**
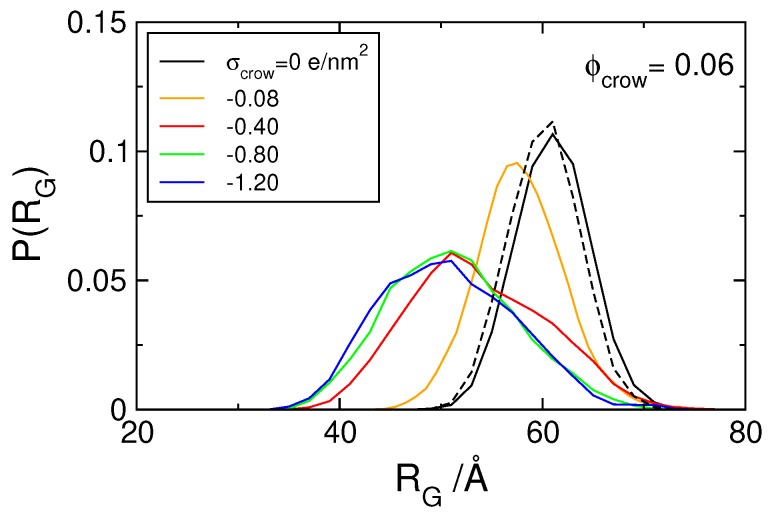
Probability distribution of the radius of gyration $P(R_{G})$, of the polyion in the absence (dashed line) and presence of crowding agents ($\Phi_{\mathrm{crow}}=$ 0.06) with varying charge density, as indicated.

We started by assessing the effect of crowding on DNA condensation in the absence of the H-NS dimers, as probed by the probability distribution of the radius of gyration, $P(R_{G})$, of the model DNA (Figure 1). The dashed and solid black curves in the figure show that the size distribution of model DNA, in the absence and presence of neutral crowding agents ($\Phi_{\mathrm{crow}}=$ 0.06), respectively, nearly overlap. In fact, and as reported in our previous study [58], no DNA condensation was observed in the presence of neutral crowders up to a volume fraction $\Phi_{\mathrm{crow}}=$ 0.20. In addition, the radius of the crowders was changed to 4, 6, and 8 Å and no significant differences were found on the size distribution of the model DNA for volume fractions of 0.16 and 0.2 (not shown). In these systems the neutral spheres are simply space filling (inert crowders) and, taking into account the moderate concentration of crowders, the polyion chain is able to maintain an extended conformation and occupy the majority of the cell.

**Figure 2:**
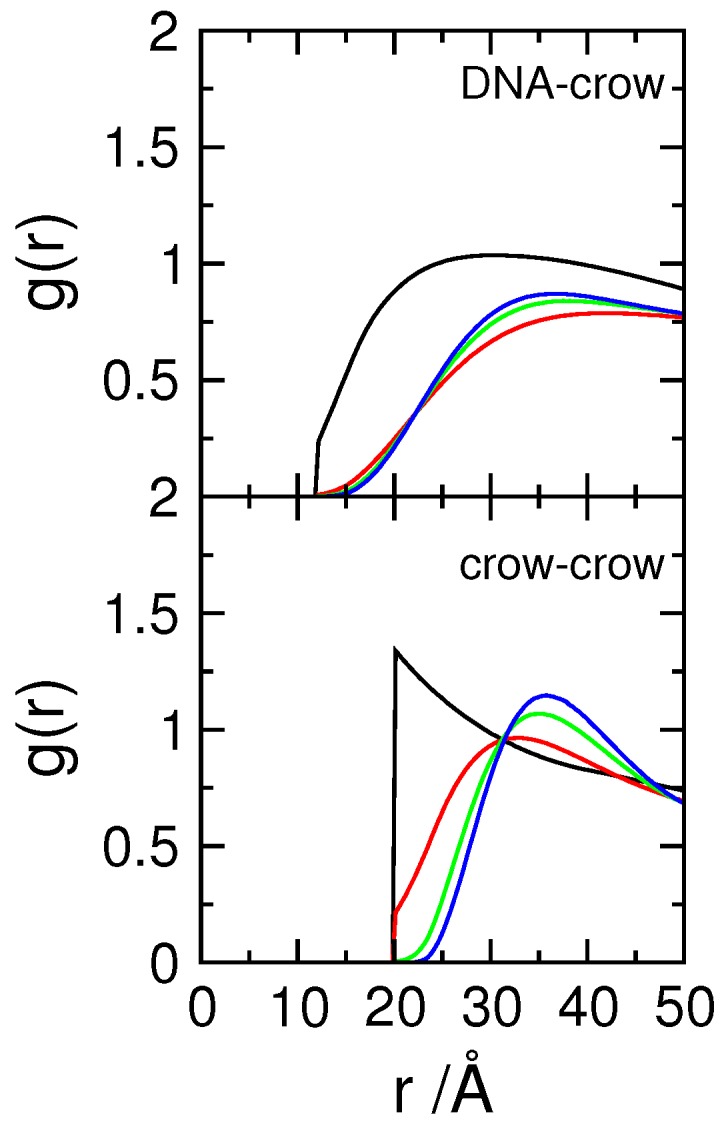
Radial distribution functions, g(r) of DNA segments-crowding agents and crowding agents-crowding agents for systems with crowding agents possessing surface charge densities, $\sigma_{\mathrm{crow}}$, of 0 (black), −0.4 (red), −0.8 (green), and −1.2 (blue) *e*/nm${}^{2}$ ($\Phi_{\mathrm{crow}}=$ 0.06).

When charged crowing agents are considered, the size distribution function of the DNA is shifted to the left, towards smaller sizes (Figure 1). Such shift begins at conditions where the crowders are monovalent, i.e., possess a very low charge density, $\sigma_{\mathrm{crow}}=$−0.08 *e*/nm${}^{2}$. An increase in the charge density on the crowders leads to a gradual shift of the size distribution (Figure 1 and Figure S2) and above $\sigma_{\mathrm{crow}}=$−0.40 *e*/nm${}^{2}$, the size distribution becomes independent of the charge density of the crowding molecules. Since the charge sign of the crowding agents is the same as the DNA (as it occurs in the bacterial cell), their interaction range is longer than that of the (neutral) hard spheres. This can be seen in the radial distributions functions of the DNA–crowders and crowders–crowders presented in Figure 2, where it is shown that the average distance between crowding molecules and that between DNA and crowding molecules increase significantly when the crowding agents are negatively charged. The average distance between the crowding agents, for example, increases from twice the radius of the particles (typical for neutral and confined particles) to nearly double that distance for the systems with the more highly charged crowders. The fact that there is no significant variation of the rdf with the crowder’s charge density above −0.8 *e*/nm${}^{2}$ indicates that the systems have reached maximum organization, promoted by the repulsive interactions between crowders. Interestingly, there is also no difference in the probability distribution of the radius of gyration of the DNA for the two systems with the higher crowding charge density, suggesting that the observed decrease in the average size of the polyanion is a consequence of the fact that the charged crowders effectively occupy, in what the DNA concerns, ‘more space’ than the corresponding neutral crowders. It should be noted that increasing the charge of the crowders increases counterion condensation and thus a decrease in the effective charge of the crowders. Based on the number of condensed ions, taken as the number of ions within a distance of 7 Å (Bjerrum length) from the surface of the crowders ($N_{\mathrm{ct}+,\;d<7\;Å}$), the effective charge of the crowders was calculated to be $Z_{\mathrm{eff}}=Z_{\mathrm{crow}}-N_{\mathrm{ct}+,\;d<7\;Å}=$−2.6, −4.5, and −6.8 *e*, for the $Z_{\mathrm{crow}}=$−5, −10, and −15 *e* ($\sigma_{\mathrm{crow}}=$−0.4–−1.2 *e*/nm${}^{2}$), respectively. Counterion condensation per se does not lead to a near constant charge of the crowders and thus is not responsible for the leveled-off condensation of DNA as the charge density of the crowders increases.

The number of counterions present in the systems varied with the surface charge density of the crowders. For system possessing crowders with the largest surface charge density, 900 counterions have been considered, which corresponds to a volume fraction of 12%. This did not, by itself, contribute to the DNA condensation, as assessed by control experiments using neutral crowders and counterions (not shown).

It should be noted that more efficient polymer condensation was obtained using similar polymer models under crowding and confinement [59]. In the mentioned work, all species were neutral and repulsive interactions were introduced between the DNA and crowders and between the DNA and confining cell. It was also found that crowding effects were stronger using crowding species smaller than the polymer beads, which mimicked a portion of DNA and associating proteins.

### 3.2. Effect of H-NS Self-Assembly on DNA Condensation

**Figure 3:**
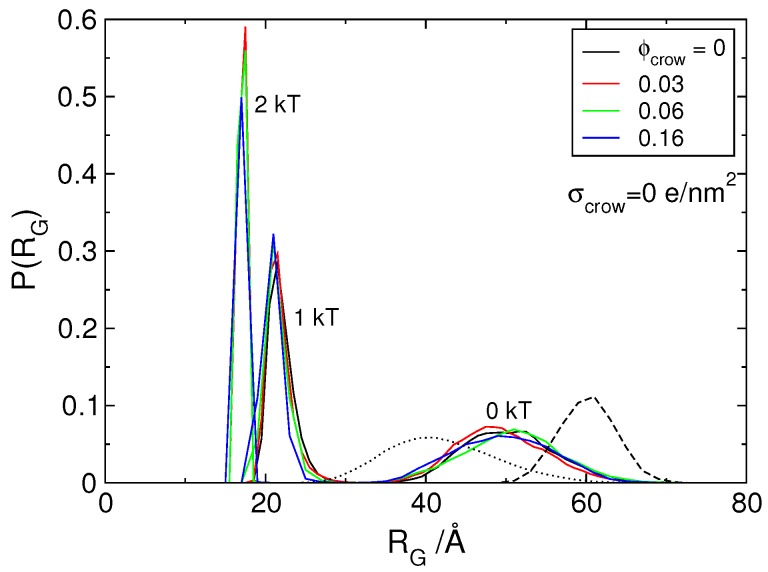
Probability distribution of the radius of gyration, $P(R_{G})$, of the polyion in the presence of model protein dimers with different attractive potential (0, 1 and 2 kT as indicated), for an increasing number of crowding particles with $\sigma_{\mathrm{crow}}=$ 0 *e*/nm${}^{2}$. The dashed line corresponds to the model DNA calculated in the presence of its counterions only and the dotted curve to the size distribution of a neutral polymer with the same number of monomers, 120.

As mentioned above, only protein concentration corresponding to a 1:1 charge ratio between protein and DNA segments was considered. The addition of H-NS dimers to DNA leads to a moderate shift of the size distribution of DNA towards smaller sizes, as seen in the dashed versus solid black curves on the right-hand side of the plot in Figure 3. The same figure shows the size distribution of a corresponding neutral chain (dotted black curve), which indicates that the presence of the dimers is not enough to neutralize the DNA chain. This is not surprising; the electrostatic interaction between the dimers and DNA is not strong enough to compensate the loss of mixing entropy of the dimers upon association to DNA, as well as the loss of conformational entropy of the DNA upon condensation [60,61].

Coincidentally, the size distribution of DNA in the presence of H-NS is very similar, in both width and average value (∼50 Å), to that of DNA in the presence of charged crowders.

It should be mentioned that other coarse-grained models have been considered for studying DNA condensation by H-NS proteins. A moderate condensation has been found using mobile DNA–protein–DNA bridges and DNA molecules with flexible regions [62]. Also, Joyeaux and co-workers have described H-NS proteins as trimers with positively charged end monomers and a negatively charged central monomer, where the protein binding rigidity and the interaction potentials have been parameterized to follow DNA condensation by cis versus trans binding of H-NS to DNA [63] and the effect of Mg${}^{2+}$ on the binding mode of H-NS to DNA [64], respectively.

One interesting characteristic of the H-NS protein is, as mentioned in the introduction, its ability to self-associate and form oligomers [46,53,54,55]. To mimic this behavior, an attractive potential was introduced between the protein segments. Figure 3 shows the $P(R_{G})$ of DNA in the presence of self-associating dimers with increasing attractive potentials (indicated in the figure), and no crowding agents (black curves). The condensation of DNA upon increasing the attractive potential from 0 to 2 kT can be clearly seen by the narrowing of the size distribution and its shift towards smaller sizes.

**Figure 4:**
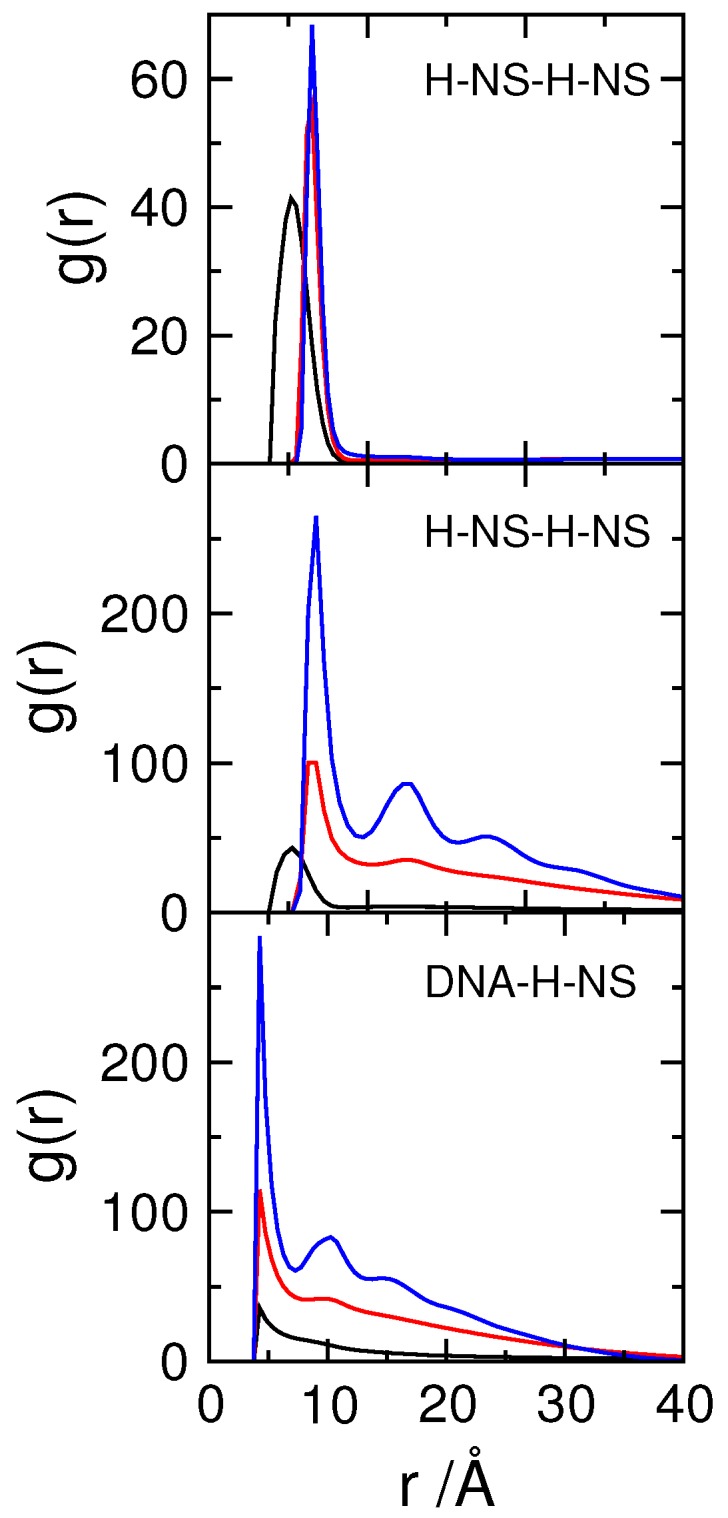
Radial distribution functions, rdf, of H-NS–H-NS segments and DNA–H-NS segments (as indicated), for systems with 60 H-NS dimers in the absence (top panel) and presence (middle and bottom panels) of DNA. The H-NS–H-NS attractive potential, $\varepsilon_{\mathrm{pp}}$, is 0 (black), 1 (red) and 2 (blue) kT. $\Phi_{\mathrm{crow}}=$ 0.

The top panel of Figure 4 shows the radial distribution functions of the H-NS monomers in the absence of DNA for the different attractive potentials, $\varepsilon_{\mathrm{pp}}=$ 0, 1, 2 kT. The introduction of $\varepsilon_{\mathrm{pp}}$ leads to the increase in the average distance between H-NS monomers in the dimers from 5.6 Å to about 6.5 Å, the distance imposed by the potential.

**Figure 5:**
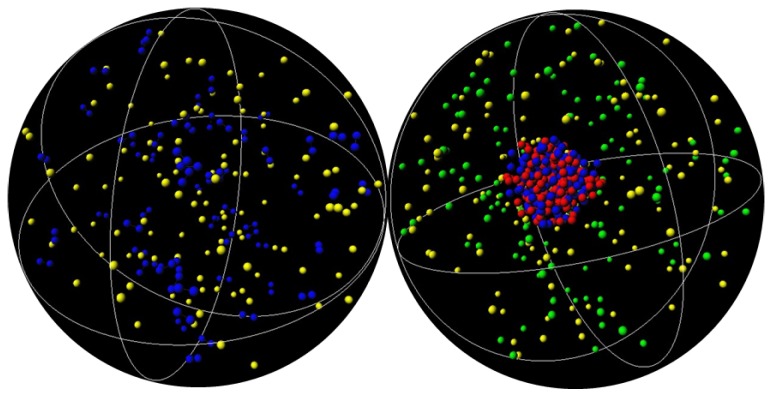
Representative snapshots showing 60 protein dimers (blue) and respective counterions (yellow) in the absence (left-hand side) and presence (right-hand side) of DNA (red) and respective counterions (green). It is clearly seen that the presence of DNA induces the association of the model H-NS proteins. $\varepsilon_{\mathrm{pp}}=$ 2 kT.

Inspection of snapshots (Figure 5) and comparison of the rdf of the H-NS–H-NS pairs in the absence (top panel) and presence (middle panel) of DNA, show that the presence of DNA enhances the association of H-NS–H-NS dimers, even for potential values that are relatively weak (1 and 2 kT). In fact, in the absence of DNA, the H-NS dimers are dispersed in solution since the attractive potential is not strong enough to overcome the electrostatic repulsion between the dimers and the decrease in their mixing entropy. The presence of the DNA chain, which has an opposite charge to that of H-NS, leads to a localized increase of the H-NS concentration in the vicinity of the DNA, favoring their self-association. This behavior resembles the association of cationic surfactants in the presence of oppositely charged polyelectrolytes [65], such as DNA [66]. The assembly of the individual proteins into an aggregate induces, in turn, the condensation of DNA, in a process that has been named double-cooperativity [66]. An increase in the H-NS–H-NS attractive potential leads to a more organized system, as attested by the appearance of more peaks in the rdfs of both H-NS–H-NS and DNA–H-NS particle pairs (middle and bottom panels in Figure 4).

Returning to the size distribution of DNA, it is also shown in Figure 3 that the presence of neutral crowding agents, up to $\Phi_{\mathrm{crow}}=$ 0.16, does not affect the conformational state of DNA in the presence of protein. However, it is interesting to note that calculations conducted with protein-to-DNA mixing ratios below charge neutrality show that crowding agents favor DNA condensation for systems with intermediate protein concentration and intermediate protein-protein association strength ($\varepsilon_{\mathrm{pp}}=$ 1 kT) [58].

### 3.3. Effect of Protein Self-Association on DNA-Protein Complex Stability

One consequence of the lack of a nuclear envelope is that there is no physical barrier that constrains the components of the nucleoid. Crowding molecules possess charged domains and their overall charge is often negative, as that of DNA, and so they can, in principle, compete for protein binding if these bind to DNA with low specificity.

**Figure 6:**
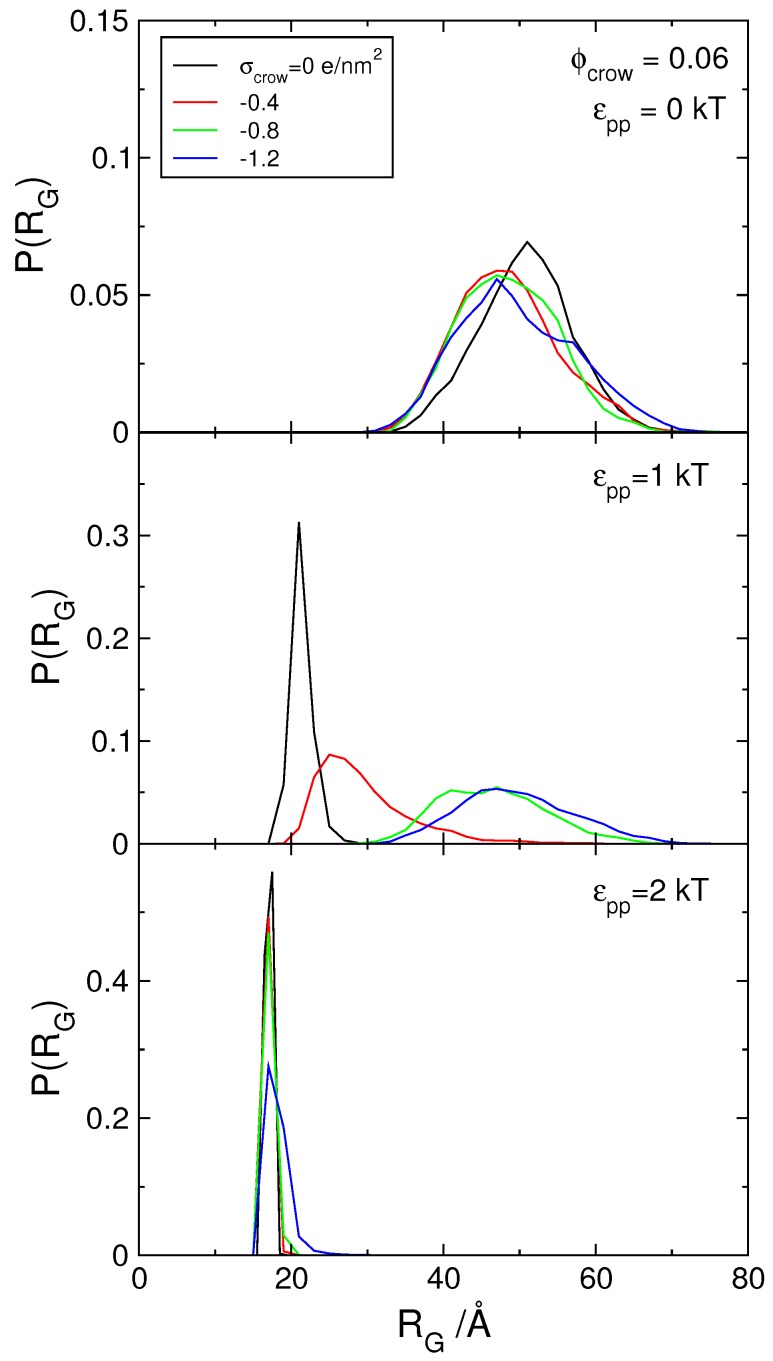
Probability distribution of the radius of gyration, $P(R_{G})$, of the polyion in the presence of model protein dimers with attractive potentials varying between $\varepsilon_{\mathrm{pp}}=$ 0 and 2 kT, in the presence of crowding molecules ($\Phi_{\mathrm{crow}}=$ 0.06) with increasing surface charge density, as indicated.

Here the effect of protein self-assembly on nucleoid stability is tested. Starting with systems where proteins do not self-associate ($\varepsilon_{\mathrm{pp}}=$ 0), it is shown (top panel in Figure 6) that increasing the charge of the crowding agents does not affect significantly the DNA size distribution, contrarily to what happened in the absence of proteins (Figure 1). This seems to suggest that the presence of charged crowding agents does not affect the DNA-protein complex, possibly due to the partial neutralization of the DNA by the proteins.

**Figure 7:**
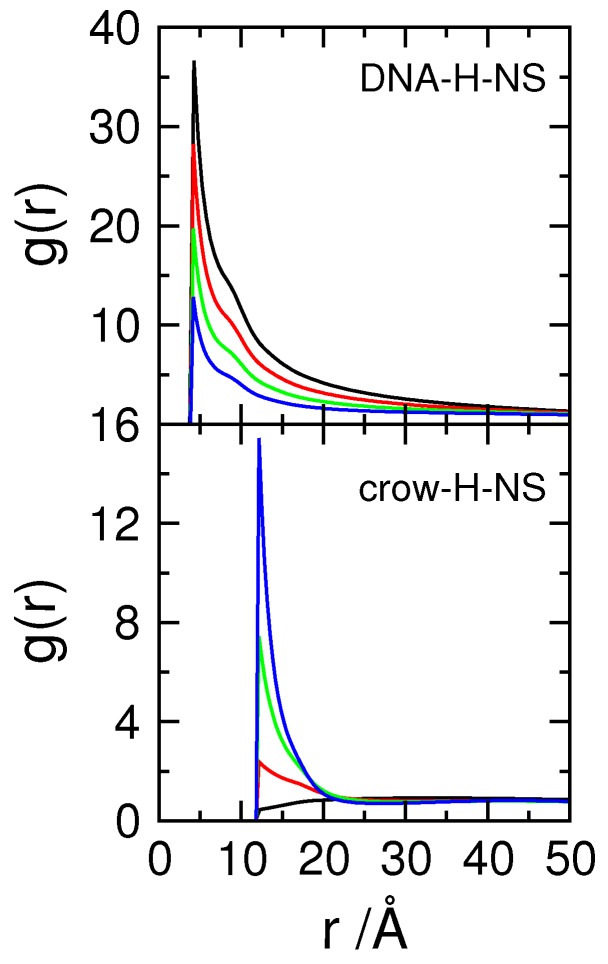
Radial distribution functions, rdf, of DNA–H-NS segments and crowding agents–H-NS segments, for systems with $\varepsilon_{\mathrm{pp}}=$ 0 kT and crowding agents ($\Phi_{\mathrm{crow}}=$ 0.06) with different charge density, $\sigma_{\mathrm{crow}}=0$ (black), −0.40 (red), −0.80 (green) and −1.20 (blue) *e*/nm${}^{2}$.

This, of course, assumes that the proteins remain associated with the DNA. Figure 7 shows the rdf for DNA–H-NS (top) and crowding particles–H-NS dimers (bottom) pairs for systems with a constant number of crowding agents ($\Phi_{\mathrm{crow}}=$ 0.06) of varying charge. When the charge density of the crowding agents increases, the number of protein dimers in contact with the DNA chain decreases (top panel). In addition, the interactions between crowding agents and H-NS dimers evolve from a hard-sphere repulsion between H-NS and neutral crowders, to an attractive interaction, depicted by the increase in the number of proteins adsorbed onto the crowding molecules with increasing (negative) surface charge density. It can be concluded that the protein dimers are partitioned between the negatively charged DNA chain and the negatively charged crowders, as would be expected from the absence of DNA–protein specific interactions. Since the DNA size distributions in the presence of solely H-NS dimers or crowding agents are very similar, the transition from one situation to the other, upon the increase in the charge density of the crowders, is not detected in the DNA size distribution analyses.

**Figure 8:**
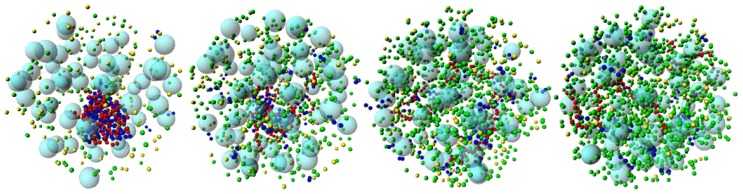
Representative snapshots showing the dissociation of the DNA (red)–H-NS (dark blue) complex upon the increase in the surface charge density of the crowding particles (light blue). The counterions of the model H-NS are represented in yellow and those of DNA and crowders in green. $\varepsilon_{\mathrm{pp}}$ = 1, $\Phi_{\mathrm{crow}}=$ 0.06 and $\sigma_{\mathrm{crow}}$ is 0, −0.40, −0.80 and −1.20 *e*/nm${}^{2}$, from the left to the right-hand side.

The middle panel in Figure 6 shows how the radius of gyration of DNA changes when the charge density of the crowding agents is increased, in the presence of proteins with weak self-association ($\varepsilon_{\mathrm{pp}}=$ 1 kT). The widening of the size distribution of DNA and its shift to larger sizes as the surface charge density of the crowding agents increases, indicate that the DNA–H-NS complex dissociates. The weak self-association between H-NS dimers gives the DNA-protein complex some stability when $\sigma_{\mathrm{crow}}$ of the crowding agents does not exceed −0.04 *e*/nm${}^{2}$ but, for crowding molecules with $\sigma_{\mathrm{crow}}\geq -$0.8 *e*/nm${}^{2}$, the complex dissociates. The snapshots in Figure 8 illustrate the dissociation of the complex and concomitant DNA decompaction as the surface charge density of the crowding agents is increased.

Interestingly, when the attractive potential between H-NS dimers is increased to 2 kT, the DNA–H-NS complex is stable even in the presence of crowding agents with the largest considered surface charge density.

**Figure 9:**
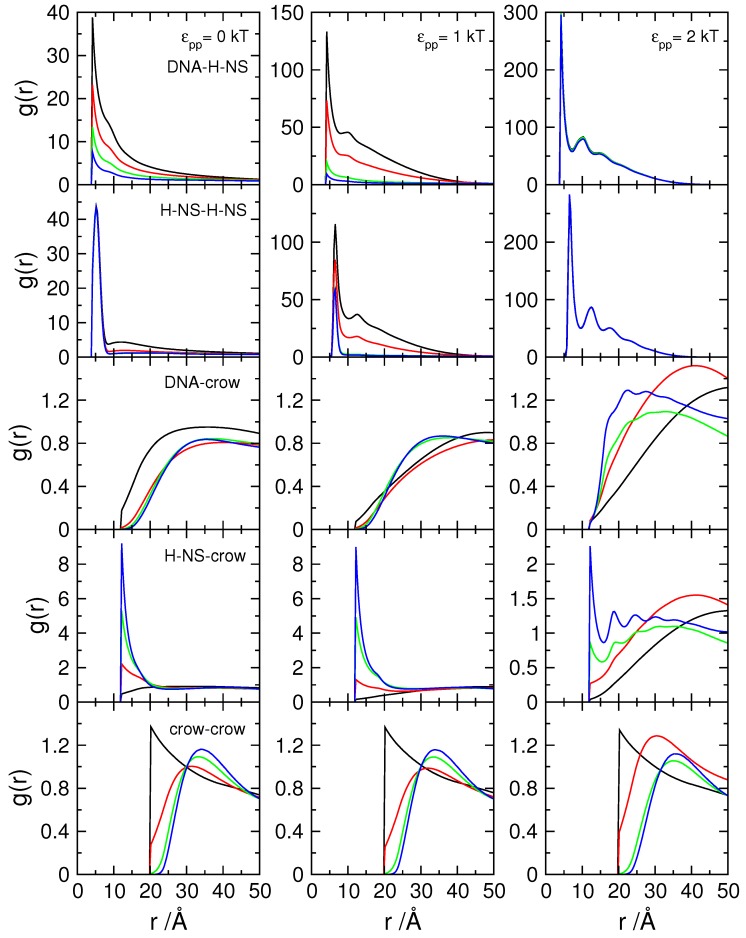
Radial distribution functions, rdf, of (from top to bottom) DNA–H-NS monomers, H-NS–H-NS monomer, DNA monomer–crowding agents, H-NS monomers–crowding particles, and crowding–crowding particles, as indicated in the panels on the left, for protein self-association potential, $\varepsilon_{\mathrm{pp}}$, of (from the left to the righ-hand-side) 0, 1, and 2 kT (as indicated in the top panels). $\Phi_{\mathrm{crow}}=$ 0.06 and $\sigma_{\mathrm{crow}}=$ 0 (black), −0.40 (red), −0.80 (green) and −1.20 (blue) *e*/nm${}^{2}$.

Figure 9 shows the rdfs of the various particle pairs in the model cell, while the results of experiments performed in the absence of DNA are shown in Figure S3. The top row in Figure 9 shows the rdfs of DNA–H-NS monomers for the three tested H-NS—H-NS attractive potentials and crowding molecules with different surface charge density, represented in different colors. The plot corresponding to the system with $\varepsilon_{\mathrm{pp}}=$ 0 kT is the same as the top panel in Figure 7, and shows, as discussed above, how the number of DNA–H-NS monomer pairs decreases when the surface charge density of the crowding molecules is increased. When an attraction potential is applied between the H-NS proteins ($\varepsilon_{\mathrm{pp}}=$ 1 kT, central column) it can be seen that, in the presence of neutral crowders (black curve), the average number of protein monomers around each DNA monomer increases. Also a second peak appears in the distribution, both indicative of protein self-association, in close resemblance to that described above in the absence of crowding molecules (bottom panel of Figure 4). When the charge density of the crowders is increased, the DNA–H-NS rdfs follow the same general behavior of the systems with no imposed attractive interaction, that is, the number of particle pairs at short separations decreases with increasing $\sigma_{\mathrm{crow}}$. This is particularly visible when $\sigma_{\mathrm{crow}}$ increases from −0.4 to −0.8 *e*/nm${}^{2}$, where the evidence of protein self-association also disappears. For $\sigma_{\mathrm{crow}}=$−1.2 *e*/nm${}^{2}$, the differences between the two sets of systems ($\varepsilon_{\mathrm{pp}}=$ 0 and 1 kT) decrease and the rdfs nearly overlap (note the different scale). This is in good agreement with the above reported competitive binding of the H-NS to the crowders (see also $g{\left(r\right)}_{H-\mathrm{NS}-\mathrm{crow}}$ in Figure 9), dissociation of the DNA–H-NS complexes, and concomitant DNA decompaction. As for the systems with $\varepsilon_{\mathrm{pp}}=$ 2 kT, there is a large increase in DNA–H-NS complex density and the complexes present a higher short-range order. In this case, increasing $\sigma_{\mathrm{crow}}$ does not affect the rdfs of DNA–H-NS particle pairs and, therefore, the integrity of the DNA–H-NS complex.

The same general trends are observed for $g{\left(r\right)}_{H-\mathrm{NS}--H-\mathrm{NS}}$ upon variations in $\varepsilon_{\mathrm{pp}}$ and $\sigma_{\mathrm{crow}}$; An increase in $\sigma_{\mathrm{crow}}$ leads to a decrease in the H-NS–H-NS monomer pairs, indicating the dissociation of the DNA–H-NS complex, with the exception of systems with $\varepsilon_{\mathrm{pp}}=$ 2 kT. It should be noted that H-NS is able to self-assemble in absence of DNA, provided that both $\varepsilon_{\mathrm{pp}}$ and $\sigma_{\mathrm{crow}}$ are sufficiently high (right-hand side panel of the top row in Figure S3). H-NS self-association takes place, in this case, at the surface of the crowding agents, as shown by the $g{\left(r\right)}_{H-\mathrm{NS}-\mathrm{crow}}$ (Figure S3) and the snapshot in Figure S4. The reason for the less efficient self-assembly of the proteins in systems without DNA may be two-fold: firstly, the surface charge density of the DNA monomers ($\sigma_{\mathrm{DNA}}=$−2.0 *e*/nm${}^{2}$) is larger than that of most highly charged crowders considered in this work; and secondly, the conformational entropy of the DNA promotes the proximity of H-NS dimers and neutralizes the H-NS–H-NS aggregates more efficiently, which reduces the electrostatic repulsions between H-NS dimers within the aggregates. Interestingly, in the absence of DNA, the protein aggregates effectively bridge a number of crowding molecules, leading to a short-range ordering of those particles, as seen by the appearance of a second peak in $g{\left(r\right)}_{\mathrm{crow}-\mathrm{crow}}$, which is not present in the systems with DNA.

Returning to Figure 9, it should be noted that the association of H-NS with crowding particle pairs increases with larger values of $\sigma_{\mathrm{crow}}$ due to the competitive binding of the proteins to the crowding molecules, as described above. For the systems with $\varepsilon_{\mathrm{pp}}=$ 2 kT, the same trend is observed but the number of particle pairs is lower than that of systems with a lower self-attractive potential (note the different scale). In addition, the series of peaks observed for the systems with higher $\sigma_{\mathrm{crow}}$ indicate a close proximity of crowding molecules and protein aggregates. However, this is not reflected in the $g{\left(r\right)}_{\mathrm{crow}-\mathrm{crow}}$, contrarily to what was observed in the systems without DNA (bottom and right-hand panel in Figure S3), and the average distance between the crowders becomes larger with increasing $\sigma_{\mathrm{crow}}$ and is roughly independent of $\varepsilon_{\mathrm{pp}}$ (bottom panels in Figure 9).

**Figure 10:**
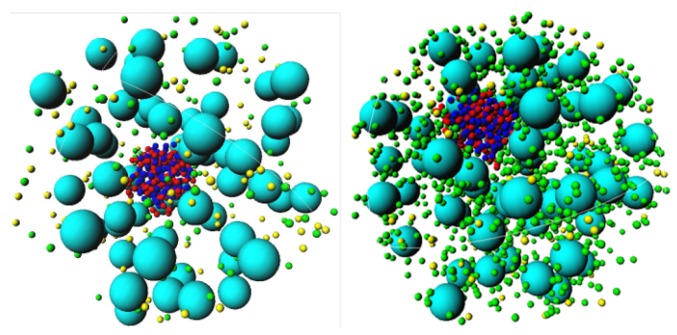
Representative snapshots showing the stability of the DNA (red)—H-NS (dark blue) complex upon the increase in the surface charge density of the crowding particles (light blue). $\varepsilon_{\mathrm{pp}}$ = 2, $\Phi_{\mathrm{crow}}=$ 0.06 and $\sigma_{\mathrm{crow}}$ is (left) 0, and (right) −1.20 *e*/nm${}^{2}$.

Regarding $g{\left(r\right)}_{\mathrm{DNA}-\mathrm{crow}}$ it is noted that, for $\varepsilon_{\mathrm{pp}}=$ 0 kT the behavior is similar to that of systems calculated in the absence of H-NS, depicted in Figure 2. Increasing the self-association potential leads to an increase in the average distance between DNA monomers and neutral crowding molecules. Interestingly, for systems with $\varepsilon_{\mathrm{pp}}=$ 2 kT and larger $\sigma_{\mathrm{crow}}$, it is observed an rdf profile consistent with weakly attractive particles. This is a consequence of the association of the DNA-protein complex to the charged crowding agents, with the H-NS aggregates promoting bridges between the DNA and the negatively crowding agents. This can also be observed in the snapshot in the right panel in Figure 10.

The preliminary results of experiments conducted in our lab suggest that while a negatively charged polymer (poly(acrylic acid)) prevents the association of cationic surfactants to DNA, leading to DNA decompaction at surfactant concentrations that would show compaction in the absence of the polyanion, negatively charged dendrimers (which are well approximated by a negatively charged sphere) do not seem to affect significantly DNA condensation by cationic surfactants. This is in good agreement with the results presented in this work. Such competition effects will be further evaluated using experimental techniques but also simulations with more realistic biological conditions.

A few final critical words on the parameters chosen for this work and the relevance of this choice on the main conclusions. The confinement of the components is a clear requirement but the size of the components is not taken to scale. In spite of crowders being smaller than one tenth of the cell, the cell size in this work was chosen so it would be big enough to assess the effect of a large number of crowders without being too computationally demanding. The fraction of crowders was, for the same reason, limited to 6%, versus the 20–30% expected for bacteria. The most significant property of the crowders in the context of this work is found to be their charge density. Increasing the concentration of crowding molecules is likely to shift the dissociation of the DNA-protein complexes towards lower protein concentrations. However, and provided that protein self-assembly is strong enough (here demonstrated for $\varepsilon_{\mathrm{pp}}=$ 2 kT), the concentration of crowders does not seem to play a significant role in the DNA-protein complex stability. As discussed above, some crowder molecules do associate with the DNA-protein complex, with the proteins serving as bridges between the DNA and the crowders (Figure 9 and Figure 10). This strongly suggests that increasing the crowder concentration will not lead to significant changes in the DNA condensation degree and DNA-protein complex stability. The overall nucleoid stabilization effect induced by protein self-assembly suggested here is thus expected to hold at larger crowding fractions and for crowders with larger surface charge densities.

The charge density and flexibility of the model DNA corresponds to a semi-flexible polymer in a good solvent but the confinement reduces the $\langle R_{g}\rangle /\langle R_{ee}\rangle$ to 3.5 ± 0.1, with $\langle R_{g}\rangle$ and $\langle R_{ee}\rangle$ the root-mean square of the radius of gyration and the end-to-end distance, respectively. It can, correctly so, be argued that DNA is a stiff molecule on a short length scale. However, increasing the rigidity of the model DNA would lead to the formation of toroids, which is less desirable in this context. A similar level of coarse-graining was chosen for the H-NS model where the charge density of H-NS binding site was taken to be equal to the DNA monomers. A thermodynamic analysis of the dimerization and tetramerization of H-NS proteins in solution (in absence of DNA) led to oligomerization enthalpy changes in the order of 10 kT at room temperature [67]. This work reports interaction energies that are lower (maximum of 2 kT) to show that even very moderate protein-protein interactions can lead to a significant stabilization of the nucleoid. Clearly, stronger protein-protein interactions, as those measured experimentally, will lead to a stronger stabilization of the DNA-protein complex. The conditions that lead to DNA–H-NS complex dissociation will be further investigated using more realistic models and biological conditions.

The counterions were taken explicitly so counterion condensation effects are, as well as other effects not accounted for by mean-field theories, included.

## 4. Conclusions

A simple coarse-grained model was used to study the effect of protein self-assembly on DNA condensation and nucleoid stability in a bacterial cell in the presence of negatively charged crowding molecules.

It is found that the presence of neutral crowding agents did not affect the condensation of DNA, up to the studied volume fraction ($\phi_{\mathrm{crow}}=$ 0.16). When negatively charged crowding agents were considered, a decrease in the average size of the DNA was observed. The addition of simple protein dimers (with a total charge matching that of the DNA) did not affect DNA condensation further since the protein dimers partitioned between the DNA and the crowding agents. However, adding a self-association potential to the protein dimers led to a significant increase in DNA condensation, even for protein–protein potentials as weak as 2 kT. The self-association of the proteins also increased the stability of DNA-protein complexes in the presence of negatively charged crowding agents.

Considering that HU, a DNA-binding protein that does not form oligomers in the cell, has been found to also bind to RNA in the ribosomes, it is proposed that the self-assembly of DNA-binding proteins not only enhances DNA condensation but also guarantees the stability of the nucleoid.

## Figures and Tables

**Table 1:** General data of the model.

Cell radius	$R_{\mathrm{cell}}=$ 100 Å
DNA monomer radius	$R_{\mathrm{mon},\mathrm{DNA}}=$ 2 Å
DNA monomer charge	$Z_{\mathrm{mon},\mathrm{DNA}}=$−1
DNA length (in no. of monomers)	$N_{\mathrm{mon},\mathrm{DNA}}=$ 120
H-NS monomer radius	$R_{\mathrm{mon},H-\mathrm{NS}}=$ 2 Å
H-NS monomer charge	$Z_{\mathrm{mon},H-\mathrm{NS}}=$ 1
H-NS length (in no. of monomers)	$N_{\mathrm{mon},H-\mathrm{NS}}=$ 2
No. of H-NS	$N_{H-\mathrm{NS}}=$ 60
Attractive potential H-NS–H-NS	$\varepsilon_{\mathrm{pp}}=$ 0–2 kT
Crowder radius	$R_{\mathrm{crow}}=$ 10 Å
Crowder surface charge density	$\sigma_{\mathrm{crow}}=$ 0–−1.2 *e*/nm${}^{2}$
Volume fraction of crowders	$\Phi_{\mathrm{crow}}=$ 0–0.06
Counterion radius	$R_{\mathrm{ct}}=$ 2 Å
Counterion charge	$Z_{\mathrm{ct}}=\pm$1
No. positively charged counterions	$N_{\mathrm{ct}+}=$ 120–1020
No. negatively charged counterions	$N_{\mathrm{ct}-}=$ 120

## Supplementary Materials

The following are available online at https://www.mdpi.com/2073-4360/11/7/1102/s1. Figure S1: Schematic representation of the used Monte Carlo trial moves, Figure S2: Size distribution of DNA in the presence of crowding agents with low charge density, Figure S3: Radial distribution functions of cell components in absence of DNA, Figure S4: Representative snapshot showing some association of the model H-NS proteins at the surface of crowding agents in absence of DNA.
