# Supplementary material for: Role of Protein Self-Association on DNA Condensation and Nucleoid Stability in a Bacterial Cell Model
Source: Polymers (Basel). 2019 Jun 29;11(7):1102. doi: 10.3390/polym11071102 (PMC6680993; doi:10.3390/polym11071102)
Supplement: Supplementary file 1 [file polymers-11-01102-s001.pdf]

Supplementary information – Role of protein self-association on DNA condensation and nucleoid stability in a bacterial cell model

Rita S. Dias*

Department of Physics, NTNU - Norwegian University of Science and Technology, NO-7491 Trondheim, Norway

E-mail: rita.dias@ntnu.no

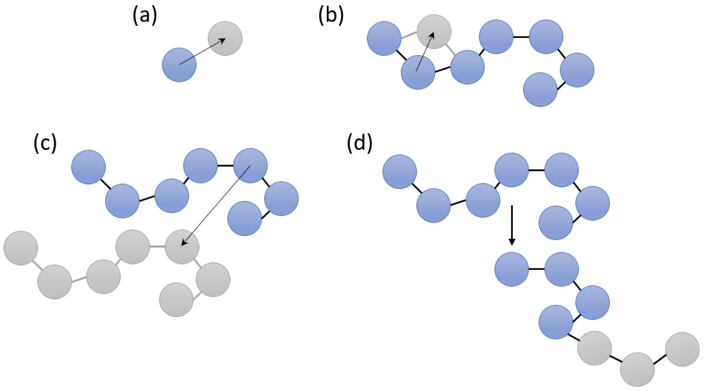

Figure S1: Schematic representation of the used Monte Carlo trial moves, depicting translational moves of (a) a single particle, (b) a single monomer within a chain, and (c) an entire chain. Panel (d) shows a slithering trial move where part of the end chain is moved to the other side of the chain with biased radial and angular position. The monomers that are part of the segment of chain that is moved keep their relative positions. The single particle move, panels (a) and (b), is attempted 100 times more often than the other types of moves.

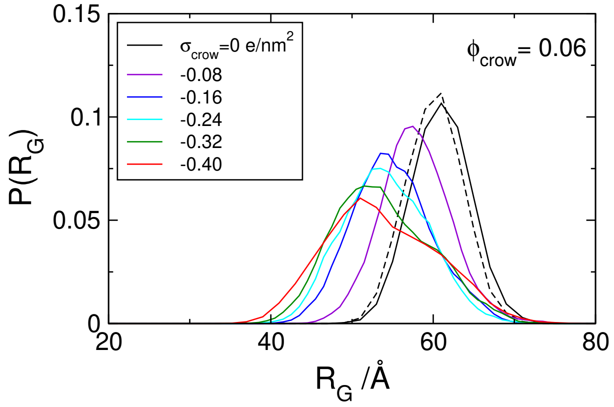

Figure S2: Probability distribution of the radius of gyration $P(R_G)$, of the polyion in the absence (dashed line) and presence of crowding agents ($\Phi_{\text{crow}} = 0.06$) possessing surface charge densities, σ_{crow}, of 0 (black), -0.08 (violet), -0.16 (blue), -0.24 (cyan), -0.32 (green) and -0.40 (red) e/nm^2, as indicated.

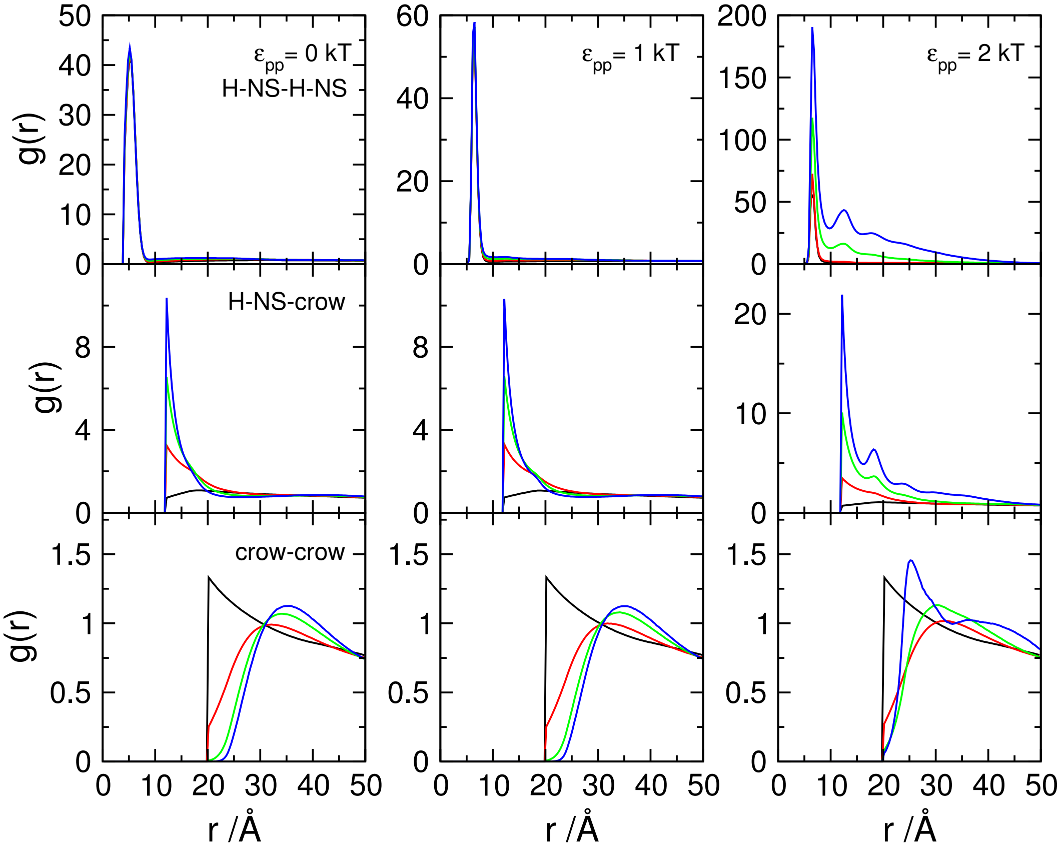

Figure S3: Radial distribution functions, rdf, of cell components in absence of DNA. From top to bottom: H-NS – H-NS monomers, H-NS monomers – crowding particles, and crowding – crowding particles, as indicated in the panels on the left, for protein self-association potential, $\varepsilon_{\mathrm{pp}}$, of (from the left to the righ-hand-side) 0, 1, and 2 kT (as indicated in the top panels). $\Phi_{\mathrm{crow}} = 0.06$ and $\sigma_{\mathrm{crow}} = 0$ (black), -0.40 (red), -0.80 (green) and -1.20 (blue) e/nm^2.

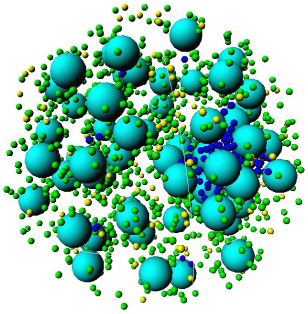

Figure S4: Representative snapshot showing some association of the model H-NS proteins (dark blue) at the surface of crowding agents (light blue) in absence of DNA. The counterions of the model H-NS are represented in yellow and those of the crowders in green. $\varepsilon_{\mathrm{pp}} = 2$ kT, $\Phi_{\mathrm{crow}} = 0.06$ and $\sigma_{\mathrm{crow}} = -1.20$ e/nm^2.
